# Sex-specific risk factors for new-onset heart failure: the PREVEND study at 25 years

**DOI:** 10.1093/eurheartj/ehae868

**Published:** 2024-12-30

**Authors:** Bart J van Essen, Johanna E Emmens, Jasper Tromp, Wouter Ouwerkerk, Marcelle D Smit, Christiane A Geluk, Lukas Baumhove, Navin Suthahar, Ron T Gansevoort, Stephan J L Bakker, Kevin Damman, Peter van der Meer, Rudolf A de Boer, Dirk J van Veldhuisen, Adriaan A Voors

**Affiliations:** Department of Cardiology, University Medical Centre Groningen, University of Groningen, Hanzeplein 1, 9713 GZ, Groningen, The Netherlands; Department of Cardiology, University Medical Centre Groningen, University of Groningen, Hanzeplein 1, 9713 GZ, Groningen, The Netherlands; Saw Swee Hock School of Public Health & The National University Health System, Singapore, Singapore; Duke-NUS Medical School, Singapore, Singapore; Department of Dermatology, Amsterdam UMC, University of Amsterdam, Amsterdam Infection and Immunity Institute, Amsterdam, The Netherlands; National Heart Research Institute Singapore, National Heart Centre Singapore, Singapore, Singapore; Department of Cardiology, Martini Hospital, Groningen, The Netherlands; Department of Cardiology, Martini Hospital, Groningen, The Netherlands; Department of Cardiology, University Medical Centre Groningen, University of Groningen, Hanzeplein 1, 9713 GZ, Groningen, The Netherlands; Erasmus MC, Cardiovascular Institute, Thorax Center, Department of Cardiology, Rotterdam, The Netherlands; Division of Nephrology, Department of Internal Medicine, University Medical Centre Groningen, University of Groningen, The Netherlands; Division of Nephrology, Department of Internal Medicine, University Medical Centre Groningen, University of Groningen, The Netherlands; Department of Cardiology, University Medical Centre Groningen, University of Groningen, Hanzeplein 1, 9713 GZ, Groningen, The Netherlands; Department of Cardiology, University Medical Centre Groningen, University of Groningen, Hanzeplein 1, 9713 GZ, Groningen, The Netherlands; Erasmus MC, Cardiovascular Institute, Thorax Center, Department of Cardiology, Rotterdam, The Netherlands; Department of Cardiology, University Medical Centre Groningen, University of Groningen, Hanzeplein 1, 9713 GZ, Groningen, The Netherlands; Department of Cardiology, University Medical Centre Groningen, University of Groningen, Hanzeplein 1, 9713 GZ, Groningen, The Netherlands

**Keywords:** Heart failure, Heart failure with preserved ejection fraction, Heart failure with reduced ejection fraction, Epidemiology, Lifetime risk

## Abstract

**Background and Aims:**

Current estimates for the lifetime risk to develop heart failure with either a reduced (HFrEF) or preserved ejection fraction (HFpEF) and their associated risk factors are derived from two studies from the USA. The sex-specific lifetime risk and population attributable fraction of potentially modifiable risk factors for incident HFpEF and HFrEF are described in a large European community-based cohort with 25 years of follow-up.

**Methods:**

A total of 8558 participants from the PREVEND cohort were studied at baseline from 1997 onwards and followed until 2022 for cases of new-onset HFrEF (ejection fraction < 50%) and HFpEF (ejection fraction ≥ 50%) by assessment of hospital records.

**Results:**

A total of 804 cases of new-onset HF were identified (534 HFrEF and 270 HFpEF) during 25 years of follow-up. The mean age at baseline was 50 years for men and 47 years for women. The mean age at onset of HF was 72.1 years in men and 74.2 years in women. The overall lifetime risk of developing HF was 24.5% in men compared to 23.3% in women. The lifetime risk of HFrEF was lower in women compared with men (11.9% vs. 18.1%), while the lifetime risk of HFpEF was higher in women compared with men (11.5% vs. 6.4%). In women, 71% of incident HFrEF cases were attributable to eight risk factors (hypertension, hypercholesterolaemia, obesity, smoking, atrial fibrillation, chronic kidney disease, myocardial infarction, and diabetes mellitus) and 60% in men. In women, 64% of incident HFpEF cases were attributable to those risk factors, whereas this was 46% in men. More specifically, in both men and women, hypertension and hypercholesterolaemia were the strongest risk factors for HFrEF, whereas hypertension and obesity were the strongest risk factors for HFpEF.

**Conclusions:**

In this European cohort, the lifetime risk of developing HFrEF was greater in men than in women, while women were at greater risk of developing HFpEF. Eight directly and indirectly modifiable risk factors substantially accounted for the risk of developing HFrEF and HFpEF, particularly in women.

## Structured Graphical Abstract

**Structured Graphical Abstract:**
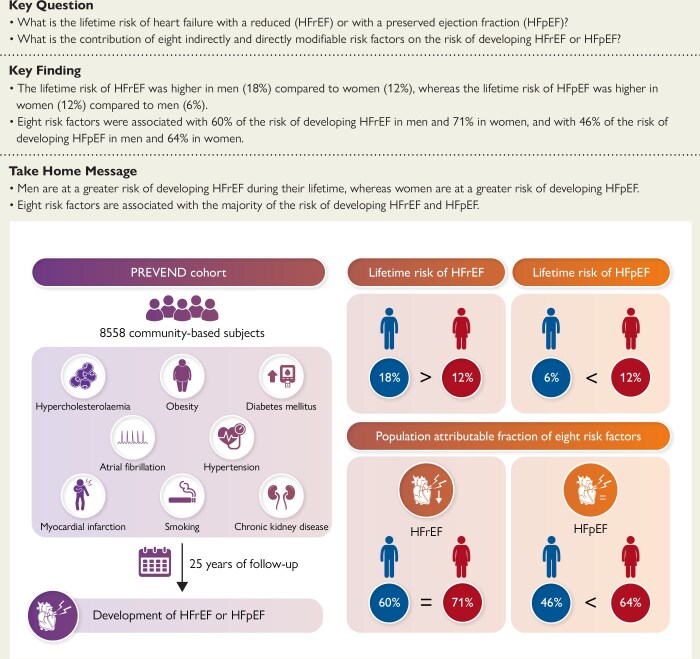
Sex-specific incidence and contribution of modifiable risk factors to new-onset heart failure with reduced and preserved ejection fraction. HFpEF, heart failure with preserved ejection fraction; HFrEF, heart failure with reduced ejection fraction; PAF, population attributable fraction; PREVEND, Prevention of REnal and Vascular ENd-stage Disease.

**See the editorial comment for this article ‘Opportunities and challenges in preventing heart failure: when is risk modifiable?’, by F. Lindberg and G. Savarese, https://doi.org/10.1093/eurheartj/ehaf042.**

## Introduction

Heart failure (HF) is emerging as the cardiovascular epidemic of the 21st century and is associated with a high mortality and morbidity. In addition, the prevalence of HF is increasing due to improvements in treatment and an ageing population. At the same time, the complexity and number of comorbidities of patients with HF are increasing, leading to higher morbidity rates including (recurrent) hospitalizations and increasing associated costs.^1,2^ Therefore, primary prevention of HF becomes increasingly important.

Several studies have deepened our understanding of incident HF, exploring a wide range of risk factors, sex differences, and pathophysiological profiles of HF with a preserved ejection fraction (HFpEF) and HF with a reduced ejection fraction (HFrEF).^3–7^ However, comprehensive studies integrating all of these elements are scarce. In addition, lifetime risk estimates of HFrEF and HFpEF were only calculated in cohorts from the USA^3,4^ and no European data on the lifetime risk of HFrEF and HFpEF are available. These previous studies have found that the incidence of HFrEF is higher in men compared with women, whereas there were conflicting results regarding the incidence of HFpEF.^3,4^ The current work is an extension of the Prevention of REnal and Vascular ENd-stage Disease (PREVEND) cohort which was previously used to investigate risk factors for HFrEF and HFpEF development.^8^ The extension of the follow-up of PREVEND allows for more reliable estimation of the lifetime risk, as well as increased statistical power in regard to the association between risk factors and the development of HFrEF and HFpEF.

In the current study, we investigated the lifetime risk and the sex-specific impact of eight directly and indirectly potentially modifiable risk factors on incident HFpEF and HFrEF. The risk factors under study are: (i) hypertension, (ii) obesity, (iii) Type 2 diabetes, (iv) hypercholesterolaemia, (v) smoking, (vi) atrial fibrillation, (vii) myocardial infarction, and (viii) chronic kidney disease (CKD). The extended follow-up of the Prevention of REnal and Vascular ENd-stage Disease (PREVEND) cohort presents a unique opportunity of a well-defined European cohort, with a follow-up duration of 25 years.

## Methods

### Study population

The present study used patient data derived from extended follow-up of the Prevention of REnal and Vascular ENd-stage Disease (PREVEND) study. The PREVEND study was a community-based study which was designed to investigate the association between micro-albuminuria (urinary albumin excretion ≥ 10 mg/L) and incident cardiorenal disease in the general population. Details about the original study design can be found elsewhere.^9^ Between 1997 and 1998, all residents aged 28–75 years of the city Groningen, The Netherlands, were approached to participate. A total of 8592 subjects were included and underwent five visits between 1997 and 2011.

### Case ascertainment and outcome data

Details about case ascertainment of HF in the period from 1997 to 2011 have been described previously.^8^ In short, all patient files from the two main hospitals in the region were screened to identify individual cases suspected of HF. Next, an endpoint adjudication committee of seven independent experts in the field of HF evaluated all cases suspected of diagnosis of new-onset HF. Each case was validated by two experts by reviewing available patient material. In case of a difference in opinion, a joint decision by the adjudication committee was made.

After 2011, HF was diagnosed by checking the patient files from the same two hospitals in Groningen. Out of the 8592 patients that were included in the original PREVEND study, 1035 moved out of the region (12%), 374 developed HF (4%), and 710 died before they developed HF (8%) at the 2011 visit. All patient records of participants who did not develop HF or did not die until 2011 were screened up until 1 January 2022. All medical records without (i) an echocardiogram, (ii) troponin or N-terminal pro natriuretic peptide (NT-proBNP) measurement, and (iii) medical correspondence by the Cardiology Department were considered free of HF. The remaining files were checked by a team of six medical doctors working in the field of cardiology to assess HF status according to the 2021 ESC Guidelines.^10^ When there was any doubt regarding the diagnosis of HF, all files were assessed by two members of an adjudication committee consisting of four HF specialists. A standardized form was used to uniformly score patients. Again, in case of difference of opinion, a joint decision by the adjudication committee was made. In our analysis, HFrEF was defined as HF with a left ventricular ejection fraction (LVEF) < 50% and HFpEF as HF with an LVEF ≥ 50%. Left ventricular ejection fraction was available in 98.3% of all HF patients, and those without were excluded from the study leaving 8558 participants in the current analysis. Mortality data were sourced from the Governmental Services from the Province of Groningen. When a participant survived, did not develop HF, and did not move from the region in the period before 2011, 1 January 2022 was used as the last date of follow-up.

### Definition of variables and data collection

Hypertension was defined as a systolic blood pressure ≥140 mmHg or a diastolic blood pressure of ≥90 mmHg or the use of antihypertensive drugs. Obesity was defined as a body mass index (BMI) of ≥30 kg/m^2^. The history of myocardial infarction was defined as evidence for myocardial infarction on baseline electrocardiogram (ECG). Type 2 diabetes was defined as a fasting plasma glucose > 7.0 mmol/L (126 mg/dL), a non-fasting plasma glucose > 11.1 mmol/L, or the use of anti-diabetic drugs. Hypercholesterolaemia was defined as total cholesterol above 5 mmol/L when there was an antecedent myocardial infarction or above 6.5 mmol/L without antecedent myocardial infarction or the use of lipid-lowering drugs. The glomerular filtration rate (eGFR) was estimated using the CKD-EPI formula.^11^ Chronic kidney disease was defined as an eGFR < 60 mL/min/1.73 m^2^. Smoking was defined as current smoking or quit smoking within the previous year. Atrial fibrillation was detected by screening of ECGs by earlier described methods.^12^ Data regarding risk factors were collected at the baseline visit between 1997 and 1998. Hypertension, obesity, Type 2 diabetes, smoking, and hypercholesterolaemia were considered directly modifiable risk factors, whereas atrial fibrillation, myocardial infarction, and CKD were considered indirectly modifiable risk factors.

### Study outcomes

The primary outcomes of the current study are (i) the lifetime risk of HF, HFpEF, and HFrEF and (ii) the population attributable fractions of eight risk factors on the development of HF, HFpEF, and HFrEF.

### Statistical analysis

Baseline characteristics were summarized by sex. Continuous data were compared using independent *t*-tests for normally distributed data, or the Mann–Whitney *U* test for non-normally distributed data. Binary data were compared with Pearson’s χ^2^ test or Fisher’s exact test dependent on the sample size.

We estimated the cumulative incidence function (CIF) for HF, HFpEF, and HFrEF while accounting death before HF and the development of the other HF subtype using the subdistribution hazard. The CIF was calculated using the *cuminc()* function from the cmprsk package in R. This approach allows for the estimation of the probability of the event of interest over time, considering the presence of competing risks that might preclude the occurrence of the event. The age of the participants was used as time-scale, rather than follow-up time in the study. The lifetime risk is defined as the cumulative incidence estimated with the subdistribution hazard of HF, HFpEF, or HFrEF up until a predefined age, and as not many participants attained an age of 90 years age or more, the cumulative age was capped at 90 years. Therefore, each participant was followed from age at entry until the date of development of HFpEF or HFrEF, censoring, death without HF, or the age of 90.

Cause-specific Cox proportional hazard models were constructed for men and women separately, and all analyses were mutually adjusted for the other risk factors. To test for potential interactions with sex models including a multiplicative interaction between sex and one of the risk factors were fitted. The model without interaction was then compared to the model with an interaction with likelihood ratio tests. Testing for interactions was done with HFpEF and HFrEF as dependent variable separately. Interaction *P*-values of <.10 were considered statistically significant. Results are presented as adjusted hazard ratios and 95% confidence intervals (95% CI). The follow-up time in the study was used as time-scale, and the proportional hazard assumption was checked using Schoenfield’s residuals and Kaplan–Meier curves. The population attributable fraction (PAF) for each risk factor was estimated using cause-specific Cox proportional hazard models and the R package graphPAF,^13^ where the formula is as follows: $\mathrm{PAF}(t)=\frac{P(T\leq t)\;-\;P(T(0)\leq t)}{P(T\leq t)}$ [*T*: time-to-event (HF); P(*T* ≤ *t*): factual probability of HF at or before time *t*; P(*T*0 ≤ *t*): counterfactual probability of HF at or before time *t* had the exposure (each risk factor) been eliminated for everyone at baseline]. PAFs for the individual risk factors were estimated in a model that also adjusted for age at baseline and for all the other risk factors. Next, the cumulative PAF was estimated, which can be interpreted as the percentage of cases that will be prevented when all eight risk factors are collectively removed from the population. The cumulative PAF is not simply the sum of the individual PAFs as one patient can have multiple risk factors. The estimation was done by earlier described methods which takes into account the causal structure of the data which is visualized in Supplementary data online, *Figure S1*.^13^ The cumulative PAF can be defined as $\mathrm{PAF}(s)=\frac{P(Y=1)\;-\;P(Y(0s)=1)}{P(Y=1)}$ [*s*: subset of risk factors, *Y*(0*s*): potential outcome where the subset of risk factors *S* has been set to their reference levels]. The procedure is described in detail under ‘Joint PAF’ in Ferguson *et al*.^13^

A sensitivity analysis was conducted by estimating the PAF for the eight risk factors for the development of HFrEF defined as an LVEF ≤ 40% and HF with mildly reduced ejection fraction (HFmrEF) (LVEF 41%–49%).

The distribution of cumulative PAFs was assumed to be normal; therefore, differences in the cumulative PAF between men and women were tested by *Z*-test. *P*-values of <.05 were considered statistically significant, except for the assessment of interactions, where *P*-values of <.10 were considered significant. All analyses were stratified for sex and performed in R version 4.3.1 (*Structured Graphical Abstract* ).^14^

## Results

### Baseline characteristics

There were 4268 men and 4290 women in the study sample. During 159 530 person-years of follow-up (median follow-up duration 23.4 years), 534 (6.2%) subjects developed HFrEF, 270 (3.2%) subjects developed HFpEF, and 1657 subjects died before they developed HF. *Table 1* presents the baseline characteristics. Men were slightly older, more often had a history of myocardial infarction (7% vs. 5%), and had a higher prevalence of hypertension (36% vs. 25%). Women more often were obese (17% vs. 14%), had a slightly lower eGFR (82 vs. 86 mL/min/1.73 m^2^), and had higher NT-proBNP at baseline (50 vs. 24 ng/L).

**Table 1:** Baseline characteristics of the Prevention of REnal and Vascular ENd-stage Disease cohort stratified by sex

	Men *N* = 4268	Women *N* = 4290	*P*-value
Age (years)	50 [40–62]	47 [38–58]	<.001
Caucasian	4028 (95.3)	4071 (95.7)	.367
BMI (kg/m^2^)	26.0 [23.8–28.4]	25.1 [22.5–28.3]	<.001
Obesity (BMI > 30 kg/m^2^)	610 (14.4)	726 (17.1)	.001
Smoking or quit <1 year	1621 (38.1)	1618 (37.8)	.798
Myocardial infarction	292 (6.9)	206 (4.9)	<.001
Hypertension	1511 (36.1)	1071 (25.3)	<.001
Hypercholesterolaemia	1178 (28.4)	1093 (26.2)	.021
Diabetes mellitus	178 (4.3)	132 (3.1)	.008
Atrial fibrillation	53 (1.3)	21 (0.5)	<.001
eGFR (mL/min/1.73 m^2^)	86 [75–96]	82 [72–92]	<.001
eGFR < 60 mL/min/1.73 m^2^	237 (5.6)	279 (6.6)	.069
NT-proBNP (ng/L)	24 [10–55]	50 [28–87]	<.001

Continuous variables are shown as median [1st–3rd quartile] and categorical data as *n* (%), and *P*-values were calculated using the Mann–Whitney *U* test for continuous data and the χ^2^ test for categorical data.

BMI, body mass index; CKD, chronic kidney disease; eGFR, estimated glomerular filtration rate; NT-proBNP, N-terminal pro-B-type natriuretic peptide.

### The lifetime risk of heart failure, heart failure with a reduced ejection fraction, and heart failure with a preserved ejection fraction

**Figure 1:**
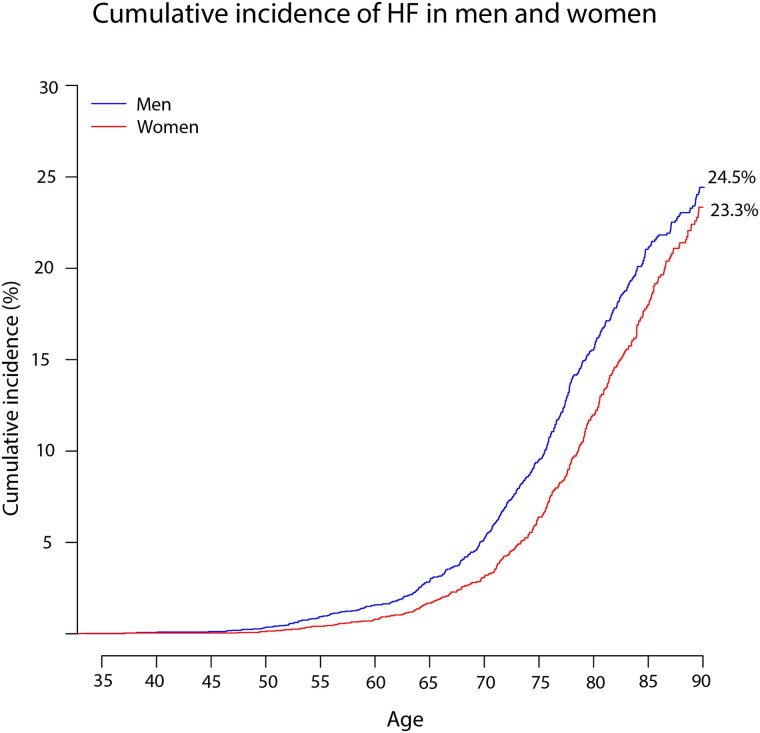
The lifetime risk of incident heart failure. The *X*-axis displays the age of the participants, and the *Y*-axis shows the cumulative incidence in percentages. The lifetime risk is the cumulative incidence of HF up until the age of 90 taking into account the competing risk of death

**Figure 2:**
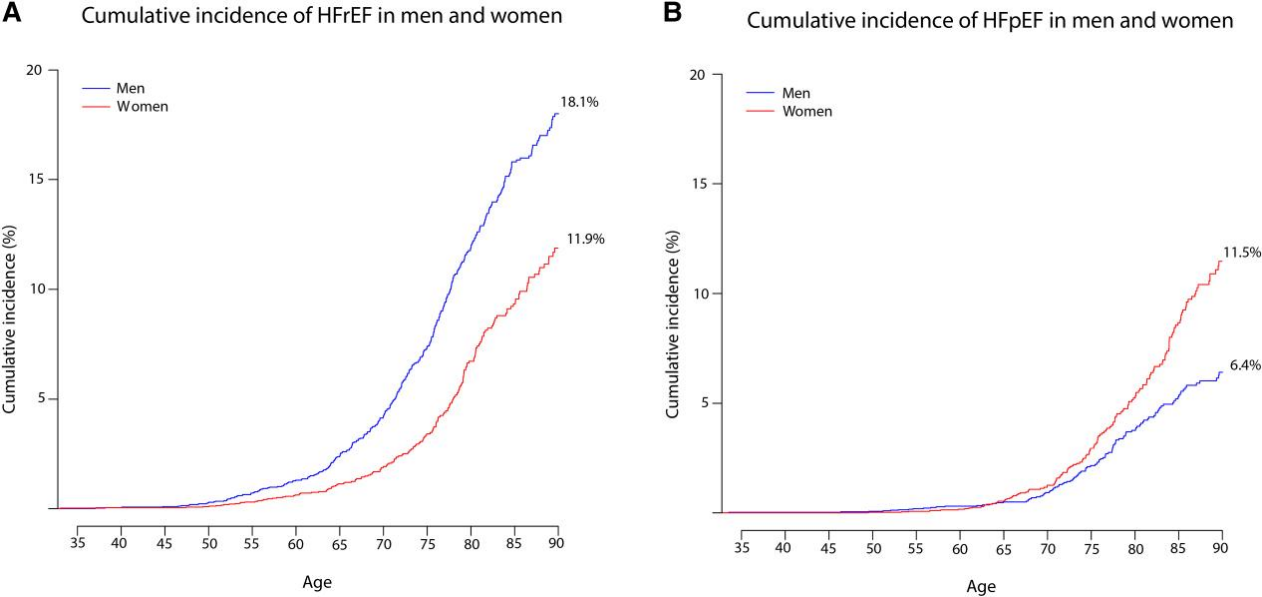
Lifetime risk of incident heart failure with reduced and heart failure with preserved ejection fraction. The *X*-axis displays the age of the participants, and the *Y*-axis shows the cumulative incidence in percentages. The lifetime risk is the cumulative incidence of heart failure up until the age of 90 taking into account the competing risk of death and the development of the other heart failure subtype

The overall lifetime risk of developing HF was 24.5% (95% CI 22.4–26.6) in men compared with 23.3% (95% CI 20.8–25.8) in women. The lifetime risk of HFrEF was 18.1% (95% CI 16.2–20.0) in men compared with 11.9% (95% CI 10.0–13.7) in women, while the lifetime risk of HFpEF was 6.4% (95% CI 5.2–7.6) in men compared with 11.5% (95% CI 9.6–13.4) in women (*Figures 1* and *2*).

The mean age of HF development was 72.1 (95% CI 71.2–73.0) years in men vs. 74.2 (95% CI 73.2–75.3) years in women. For HFrEF, the mean age of onset was 71.6 (95% CI 70.5–72.6) years in men vs. 72.9 (95% CI 71.3–74.4) years in women. For HFpEF, the mean age of onset was 73.8 (95% CI 72.0–75.6) years in men, vs. 75.8 (95% CI 74.4–77.1) years in women.

### Risk factors for the development of heart failure with a preserved ejection fraction and heart failure with a reduced ejection fraction

In men, hypertension, hypercholesterolaemia, obesity, myocardial infarction, atrial fibrillation, diabetes, and smoking were associated with the development of HFrEF. Whereas in women, hypertension, hypercholesterolaemia, myocardial infarction, atrial fibrillation, and smoking were significantly associated with the development of HFrEF.

In men, hypertension, obesity, diabetes, and atrial fibrillation were significantly associated with the development of HFpEF. Whereas in women, hypertension, smoking, obesity, and myocardial infarction were significantly associated with incident HFpEF. There was a significant interaction between diabetes and sex, where diabetes was a stronger risk factor for incident HFpEF in men compared with women (*P*-value .070). The results are summarized in Supplementary data online, *Table S1* and *Figure S2*.

### Population attributable fractions

#### Heart failure with a reduced ejection fraction

In men, the cumulative population attributable fraction of all eight potentially modifiable risk factors for HFrEF was 60%. The highest proportion (23%) was attributable to hypertension, followed by 20% to hypercholesterolaemia, 18% to myocardial infarction, 16% to smoking, and 10% to obesity. In women, the cumulative population attributable risk was 71%. This cumulative risk was largely attributable to hypertension (39%), hypercholesterolaemia (28%), and smoking (19%). Obesity, myocardial infarction, atrial fibrillation, and diabetes explained less of the risk with PAFs of, respectively, 6%, 10%, 10%, and 5%. The absolute difference of cumulative population attributable fraction between men and women for HFrEF was 11% (95% CI −2–23%, *P* = .062).

#### Heart failure with a preserved ejection fraction

**Figure 3:**
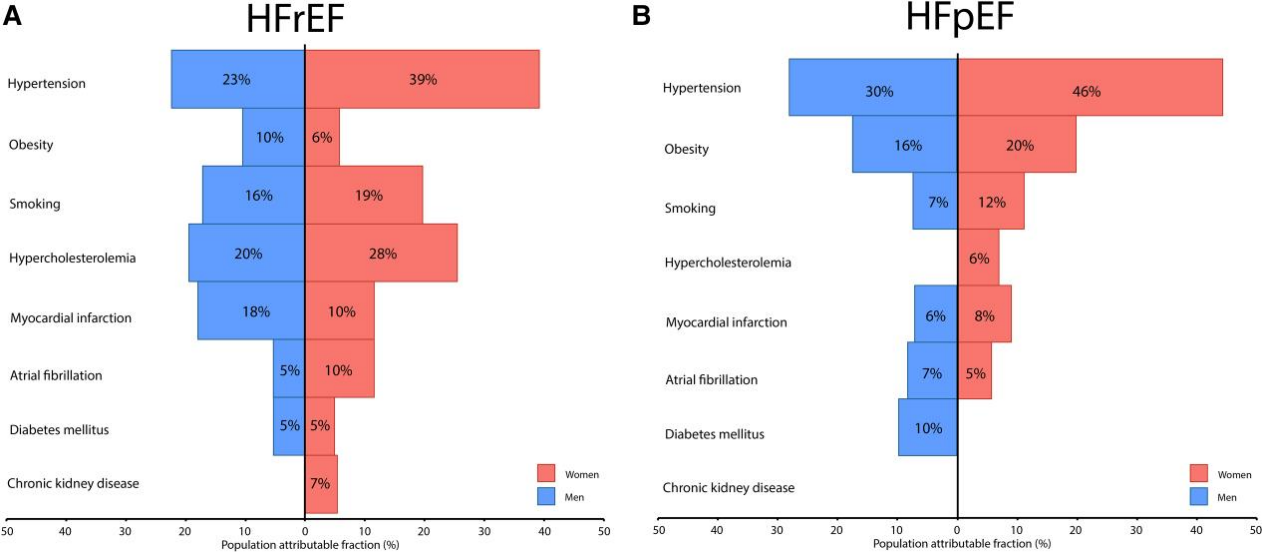
Population attributable fractions of eight comorbidities for heart failure with reduced and heart failure with preserved ejection fraction. Population attributable fraction estimation was done using multivariable-adjusted cause-specific Cox proportional hazard models adjusted for the other seven comorbidities and age at baseline, and separate models were fitted for men and women. Population attributable fractions are shown using heart failure with reduced ejection fraction as dependent variable on the left (*A*) and heart failure with preserved ejection fraction as dependent variable on the right (*B*)

In men, the cumulative attributable fraction of all eight potentially modifiable risk factors was 46%. Hypertension was the strongest risk factor (30%) followed by obesity (16%) and diabetes (10%). The other risk factors contributed less (CKD −6%, hypercholesterolaemia −1%, myocardial infarction 6%, atrial fibrillation 7%, and smoking 7%). In women, the cumulative population attributable fraction for HFpEF was 64%. This was largely driven by hypertension and obesity (46% and 20%, respectively). For hypercholesterolaemia, smoking, myocardial infarction, atrial fibrillation, diabetes mellitus, and CKD, the PAFs, respectively, were 6%, 12%, 8%, 5%, −2%, and −6%. The absolute difference of cumulative population attributable fraction between men and women for HFpEF was 19% (95% CI 6%–31%, *P* = .049). The results are summarized in *Figure 3* and *Table 2*.

**Table 2:** Population attributable fractions for heart failure with reduced ejection fraction and heart failure with preserved ejection fraction

	HFrEF, PAF (95% CI)			HFpEF, PAF (95% CI)		
	Men	Women	*P* (difference men/women)	Men	Women	*P* (difference men/women)
Hypertension	0.23 (0.09–0.37)	0.39 (0.20–0.57)		0.30 (0.06–0.55)	0.46 (0.28–0.64)	
Hypercholesterolaemia	0.20 (0.11–0.28)	0.28 (0.12–0.43)		−0.01 (−0.20–0.18)	0.06 (−0.16–0.28)	
Obesity	0.10 (0.04–0.15)	0.06 (−0.06–0.18)		0.16 (0.04–0.28)	0.20 (0.06–0.34)	
Myocardial infarction	0.18 (0.11–0.25)	0.10 (0.01–0.18)		0.06 (−0.04–0.16)	0.08 (−0.01–0.18)	
Atrial fibrillation	0.05 (0.01–0.09)	0.10 (−0.01–0.21)		0.07 (−0.03–0.17)	0.05 (−0.07–0.17)	
Chronic kidney disease	−0.01 (−0.06–0.05)	0.07 (−0.03–0.17)		−0.06 (−0.20–0.08)	−0.06 (−0.18–0.04)	
Diabetes mellitus	0.05 (0.00–0.09)	0.05 (−0.01–0.10)		0.10 (0.00–0.19)	−0.02 (−0.07–0.03)	
Smoking	0.16 (0.08–0.24)	0.19 (0.07–0.31)		0.07 (−0.08–0.23)	0.12 (0.02–0.21)	
Cumulative PAF	0.60 (0.53–0.68)	0.71 (0.63–0.80)	.062	0.46 (0.30–0.61)	0.64 (0.54–0.74)	.049

Population attributable fraction estimation was done using multivariable-adjusted cause-specific Cox proportional hazard models adjusted for the other seven comorbidities and age at baseline, and separate models were fitted for men and women. Population attributable fractions are shown using HFrEF as dependent variable on the left and HFpEF as dependent variable on the right. The cumulative PAF was estimated following earlier described methods and is not simply the sum of the individual PAFs.

Population attributable fractions for HFrEF and HFpEF without stratification for sex were performed, and the results can be found in Supplementary data online, *Figure S3*.

### Sensitivity analysis

During the total follow-up of the PREVEND cohort, 178 patients developed HFmrEF (LVEF 41%–49%). Hypertension, obesity, smoking, and hypercholesterolaemia emerged as the main risk factors for HFrEF (LVEF ≤ 40%) in men and women. For HFmrEF, hypertension, hypercholesterolaemia, myocardial infarction, and smoking emerged as the strongest risk factors. The results are summarized in Supplementary data online, *Table S2*.

## Discussion

One of the key findings of this study is that one out of four subjects will develop HF in life, with a similar risk between men and women. However, men are at greater risk for incident HFrEF and develop HF earlier in life, while women are at greater risk for incident HFpEF and develop HF later in life. The current estimates for the lifetime risk of HFrEF and HFpEF are all based on studies from the USA. To the best of our knowledge, these are the first European estimates of the lifetime risk of HFrEF and HFpEF. In addition, we found that eight directly and indirectly modifiable risk factors, and especially hypertension and obesity, are associated with the majority of the risk of incident HFrEF and HFpEF. Those risk factors, and hypertension in particular, confer a greater risk in women than in men. Collectively, our data suggest that proactive screening and treatment of eight modifiable risk factors on a population level may have the potential to substantially reduce the risk of developing HF.

These data extend our previous analyses in the PREVEND cohort.^5,6,8^ Brouwers *et al*.^8^ described the risk factors and precipitants of HFrEF and HFpEF, and Tromp and Suthahar described sex- and age-specific aspects of incident HF in joint analyses of PREVEND with FHS, CHS, and MESA.^5,6^ However, follow-up in these studies was on an average 11 years, and the cumulative incidence of HF across the life course could only be studied until a certain age. In this analysis, we were able to extend the follow-up up to 25 years, which allowed us to make more reliable lifetime risk estimates. In addition, the extended follow-up led to the collection of more heart failure events, thereby increasing statistical power for assessing the association between risk factors and HF.

The finding that ∼24% of men and 23% of women develop HF during their lifetime is consistent with data from the Framingham Heart Study, which found similar estimates.^3^ However, studies examining lifetime risk that distinguish between HFrEF and HFpEF are scarce, and only two studies to date have reported this.^3,4^ A pooled analysis of the Cardiovascular Health Study and the Multi-Ethnic Study of Atherosclerosis (CHS/MESA) found that the lifetime risk of HFrEF was higher in men than in women, whereas the lifetime risk of HFpEF was similar in both sexes.^4^ This contrasts with our finding that the incidence of HFpEF is higher in women than in men. A possible explanation could be that a different classification of HF was used (HFpEF LVEF > 45%), thus including patients in the HFpEF group who are traditionally classified as having HF with HFmrEF. As HFmrEF is more common in men,^15^ this may balance out the sex differences in the incidence of HFpEF. The results from the other study investigating the lifetime risk of HFrEF and HFpEF are consistent with our results, as they found that the incidence of HFpEF was higher in women than in men.^3^

Our study showed that eight directly and indirectly potentially modifiable risk factors are associated with the majority of the risk of incident HFpEF and HFrEF. These results convey a positive message, namely that the prevention and treatment of well-known risk factors may have the potential to substantially reduce cases of incident HF. Not many studies calculated attributable risk for HFrEF and HFpEF separately; however, one other study by Tromp *et al*.^6^ also found that the majority of HF risk is attributable to seven risk factors. In addition, data about the attributable risk of individual risk factors are important to guide public-health decisions, especially since the global prevalence of those risk factors is increasing. As attributable risk is influenced by risk factor prevalence, it is expected that in populations with different risk factor distributions than the PREVEND cohort, the attributable risk will be different. Since 1990, the number of people with hypertension worldwide has doubled, whereas the number of people with obesity has tripled.^16,17^ Consequently, population attributable fractions are anticipated to rise for risk factors with increasing prevalence; however, this may be counterbalanced by improved treatment of these risk factors, leading to a reduction in relative risk. Hypertension and obesity emerged as the main risk factors of HFpEF in men and women, whereas hypertension and hypercholesterolaemia were risk factors of HFrEF. Our current results together with the increasing prevalence of those risk factors should prompt policymakers to get effective risk factors treatment and prevention programmes in place to prevent the looming pandemic of HF cases.

The results of our analysis suggest that the joint contribution of the eight risk factors contributes more to the development of HFpEF in women compared with men. This is supported by recent studies, including an analysis in PREVEND, showing that hypertension is a risk factor for HF in general, but stronger in women compared with men.^18,19^ In addition, hypercholesterolaemia was found to be a risk factor for HFrEF in men and women; however, it was a stronger risk factor in women than in men. It remains to be determined whether these differences are attributable specifically to that risk factor or also to risk factor underdiagnosis and treatment in women. The latter is supported by the results of a study that found that lipid-lowering therapy goals were less likely to be achieved by women, pointing more towards sex differences in reaching treatment goals.^20^ It also strongly underscores the notion that tailored sex-specific preventative strategies are warranted, with a special focus on blood pressure and lipid-spectrum management in women. Another interesting explanation for a lower cumulative PAF for HFpEF in men is that other risk factors than the ones currently included in the model are associated with the development of HFpEF in men. Future studies should elucidate which other risk factors are associated with the development of HFpEF in men.

In sensitivity analysis, we have found that HFrEF (LVEF ≤ 40%) and HFmrEF (LVEF 41%–49%) have many shared risk factors displayed by the importance of hypertension, hypercholesterolaemia, myocardial infarction, and smoking. This was also found in recent literature in which HFmrEF was found to be more similar to HFrEF than HFpEF in regard to treatment response and clinical characteristics (in particular the presence of ischaemic aetiology).^15^ It must be noted that the HFmrEF group was small, which limited the statistical power for some risk factors (for example, atrial fibrillation).

The strengths of this study include the long-term follow-up of the PREVEND cohort, together with a manual workflow for the diagnosis of HF. All potential HF cases were evaluated by either a physician or a panel of experts in the case of doubt. The risk of misclassification of the outcome is therefore minimal. Another strength of this study is that patients were not only included when they were admitted to the hospital but also when they presented to the outpatient clinic. It is important to note that patients at the general practitioner were not included in the study. There are also several limitations that need to be addressed. First of all, the PREVEND study consists of participants mostly from Caucasian origin, thereby limiting the external generalizability of this study. Second, in the period from 2011 to 2022, no data about migration were available, which might have led to an under-detection of HF cases. The impact is however expected to be minor: the lifetime risk estimates in our study are similar to estimates from the other large cohort studies investigating HFrEF and HFpEF incidence. Third, the PREVEND study oversampled participants with micro-albuminuria, which might lead to a population with a higher baseline risk of developing HF. Fourth, myocardial infarction was diagnosed by the presence of ECG changes at baseline; therefore, prior non-ST elevation myocardial infarction might not have been detected, potentially leading to an underestimation of the PAF associated with myocardial infarction. Fifth, only baseline risk factors were included in the current study, risk factors that might have developed over time were not considered. This might lead to non-differential misclassification of one or more of the risk factors in which a bias towards the null is expected. Sixth, most of the participants of PREVEND were younger than 90 years of age at the end of follow-up, which might introduce uncertainty regarding our lifetime risk estimates. The lifetime risk estimates of this study do however align with previous studies regarding the lifetime risk of HFrEF and HFpEF.^3,4^

## Conclusions

In this large, population-based cohort study from Europe, the incidence of HFrEF was higher in men, while the risk of incident HFpEF was higher in women. Additionally, eight indirectly and directly modifiable risk factors were associated with the majority of the risk for both types of HF. These risk factors contributed more to the risk of developing HFpEF in women than in men. In conclusion, these findings support the notion that proactive screening of modifiable risk factors may have the potential to substantially reduce the incidence of HF, in particular in women.

## Supplementary Material

ehae868_Supplementary_Data

## Declarations

### Data Availability

The data are available upon reasonable request.
