# Supplementary material for: Sex-specific risk factors for new-onset heart failure: the PREVEND study at 25 years
Source: Eur Heart J. 2024 Dec 30;46(16):1528–36. doi: 10.1093/eurheartj/ehae868 (PMC12011521; doi:10.1093/eurheartj/ehae868)
Supplement: ehae868_Supplementary_Data [file ehae868_supplementary_data.docx]

**Supplementary Material**

**Supplementary Table 1: Coefficients of cause-specific Cox regression models.**

|  | **HFrEF** | | | | | **HFpEF** | | | | |
| --- | --- | --- | --- | --- | --- | --- | --- | --- | --- | --- |
|  | **Men** | | **Women** | | **p interaction** | **Men** | | **Women** | | **p interaction** |
|  | **HR (95% CI)** | **p** | **HR (95% CI)** | **p** |  | **HR (95% CI)** | **p** | **HR (95% CI)** | **p** |  |
| **Hypertension** | 1.51  (1.18-1.92) | 0.001 | 2.25  (1.58-3.21) | <0.001 | 0.100 | 1.67  (1.09-2.57) | 0.020 | 2.55  (1.74-3.74) | <0.001 | 0.170 |
| **Hypercholesterolemia** | 1.55  (1.22-1.97) | <0.001 | 1.70  (1.21-2.39) | 0.002 | 0.783 | 0.92  (0.59-1.42) | 0.701 | 1.12  (0.78-1.61) | 0.541 | 0.362 |
| **Obesity** | 1.65  (1.26-2.14) | <0.001 | 1.25  (0.88-1.76) | 0.213 | 0.371 | 2.11  (1.36-3.27) | 0.001 | 1.86  (1.31-2.64) | <0.001 | 0.716 |
| **Myocardial infarction** | 2.67  (2.00-3.57) | <0.001 | 1.96  (1.23-3.12) | 0.004 | 0.451 | 1.69  (0.86-3.30) | 0.126 | 1.92  (1.11-3.32) | 0.019 | 0.466 |
| **Atrial fibrillation** | 2.85  (1.70-4.77) | <0.001 | 7.24  (2.79-18.76) | <0.001 | 0.162 | 3.60  (1.53-8.45) | 0.003 | 4.03  (0.96-16.85) | 0.056 | 0.817 |
| **Chronic kidney disease** | 1.01  (0.70-1.44) | 0.964 | 1.37  (0.91-2.06) | 0.130 | 0.143 | 0.68  (0.33-1.39) | 0.292 | 0.73  (0.44-1.21) | 0.224 | 0.686 |
| **Diabetes mellitus** | 1.62  (1.08-2.42) | 0.019 | 1.65  (1.00-2.74) | 0.051 | 0.979 | 2.22  (1.14-4.33) | 0.019 | 0.84  (0.42-1.66) | 0.611 | 0.070 |
| **Smoking** | 1.63  (1.30-2.05) | <0.001 | 1.97  (1.42-2.74) | <0.001 | 0.662 | 1.23  (0.80-1.87) | 0.345 | 1.52  (1.05-2.21) | 0.027 | 0.598 |

**Supplementary Table 2: sensitivity analysis of population attributable fractions for eight risk factors for HFrEF (LVEF <40%) and HFmrEF (LVEF 41 – 49%).**

|  | **HFrEF (LVEF <40%)** | | **p**  **(difference men/women)** | **HFmrEF (LVEF 41 – 49%)** | | **p**  **(difference men/women)** |
| --- | --- | --- | --- | --- | --- | --- |
|  | **Men** | **Women** |  | **Men** | **Women** |  |
|  | **PAF (95% CI)** | **PAF (95% CI)** |  | **PAF (95% CI)** | **PAF (95% CI)** |  |
| **Hypertension** | 0.19  (0.04-0.34) | 0.31  (0.08-0.54) |  | 0.33  (0.10-0.57) | 0.50  (0.29-0.72) |  |
| **Hypercholesterolemia** | 0.19  (0.08-0.30) | 0.25  (0.06-0.43) |  | 0.22  (0.07-0.38) | 0.34  (0.07-0.62) |  |
| **Obesity** | 0.11  (0.05-0.17) | 0.11  (-0.07-0.3) |  | 0.07  (-0.05-0.19) | -0.01  (-0.17-0.15) |  |
| **Myocardial infarction** | 0.22  (0.13-0.30) | 0.09  (-0.01-0.19) |  | 0.08  (-0.03-0.19) | 0.14  (-0.05-0.33) |  |
| **Atrial fibrillation** | 0.06  (0.01-0.10) | 0.05  (-0.07-0.17) |  | 0.05  (-0.01-0.11) | 0.20  (-0.05-0.44) |  |
| **Chronic kidney disease** | 0.00  (-0.06-0.07) | 0.12  (0.00-0.25) |  | -0.03  (-0.14-0.08) | -0.01  (-0.15-0.13) |  |
| **Diabetes mellitus** | 0.03  (-0.02-0.08) | 0.06  (-0.02-0.13) |  | 0.10  (0.00-0.19) | 0.03  (-0.06-0.12) |  |
| **Smoking** | 0.15  (0.06-0.25) | 0.20  (0.05-0.35) |  | 0.18  (0.01-0.34) | 0.16  (-0.01-0.34) |  |
| **Cumulative PAF** | 0.59  (0.52-0.69) | 0.69  (0.52-0.91) | 0.322 | 0.61  (0.44-0.77) | 0.73  (0.55-0.91) | 0.319 |

**Supplementary Figure 1: causal diagram relating the eight risk factors to heart failure development.**


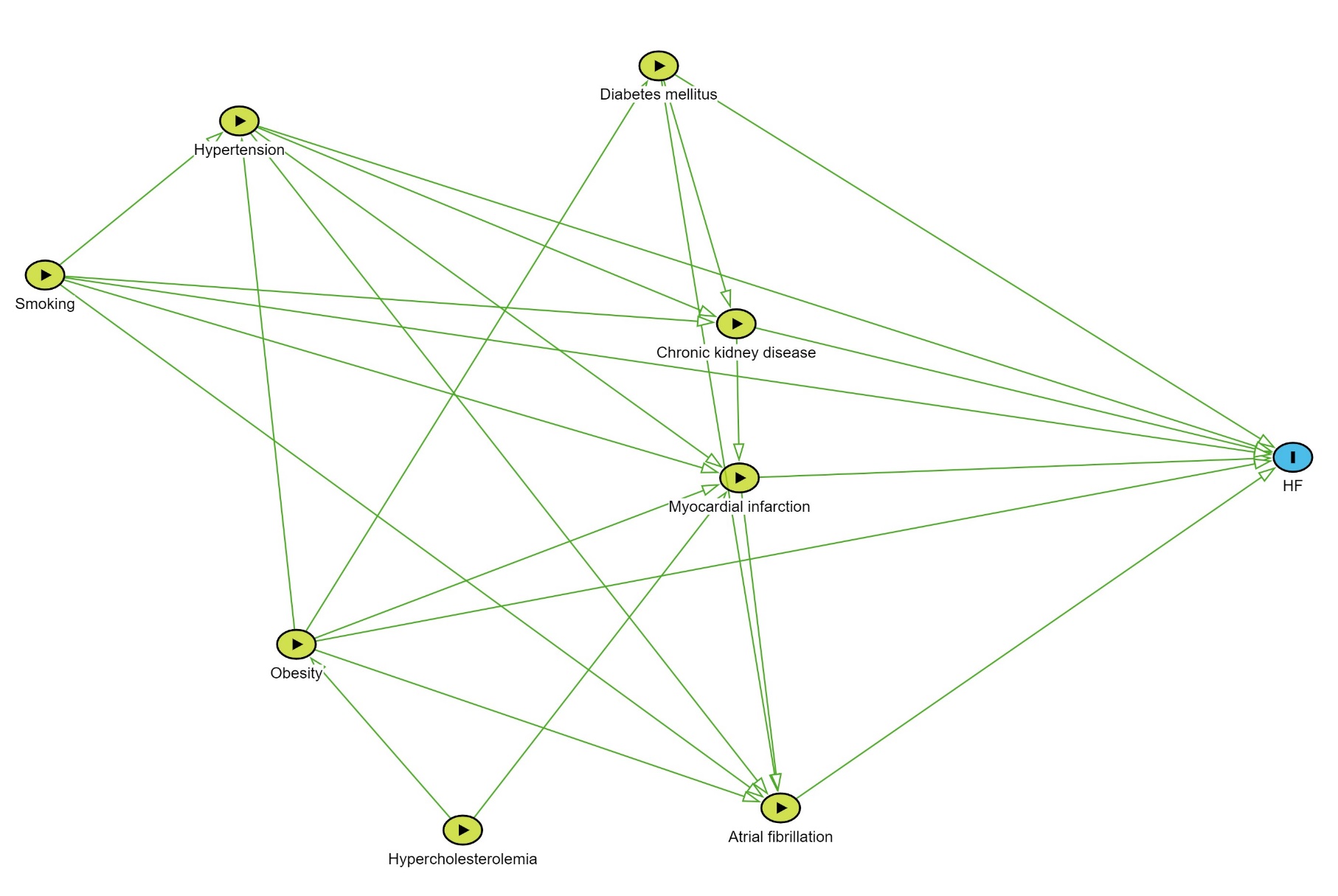


**Supplementary Figure 2: Forest Plot for coefficients of cause-specific Cox proportional hazard models for the association between risk factors and HFpEF and HFrEF. Left association of risk factors with HFrEF, right association of risk factors with HFpEF.**

**
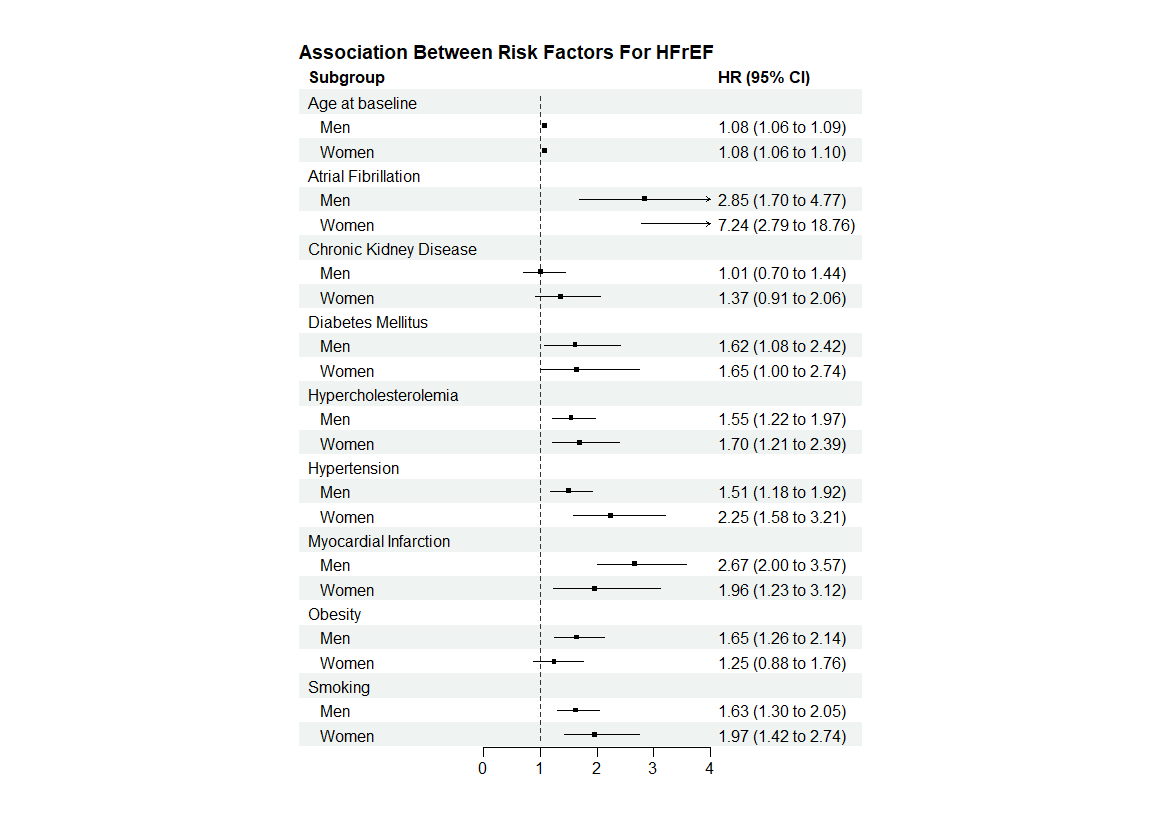

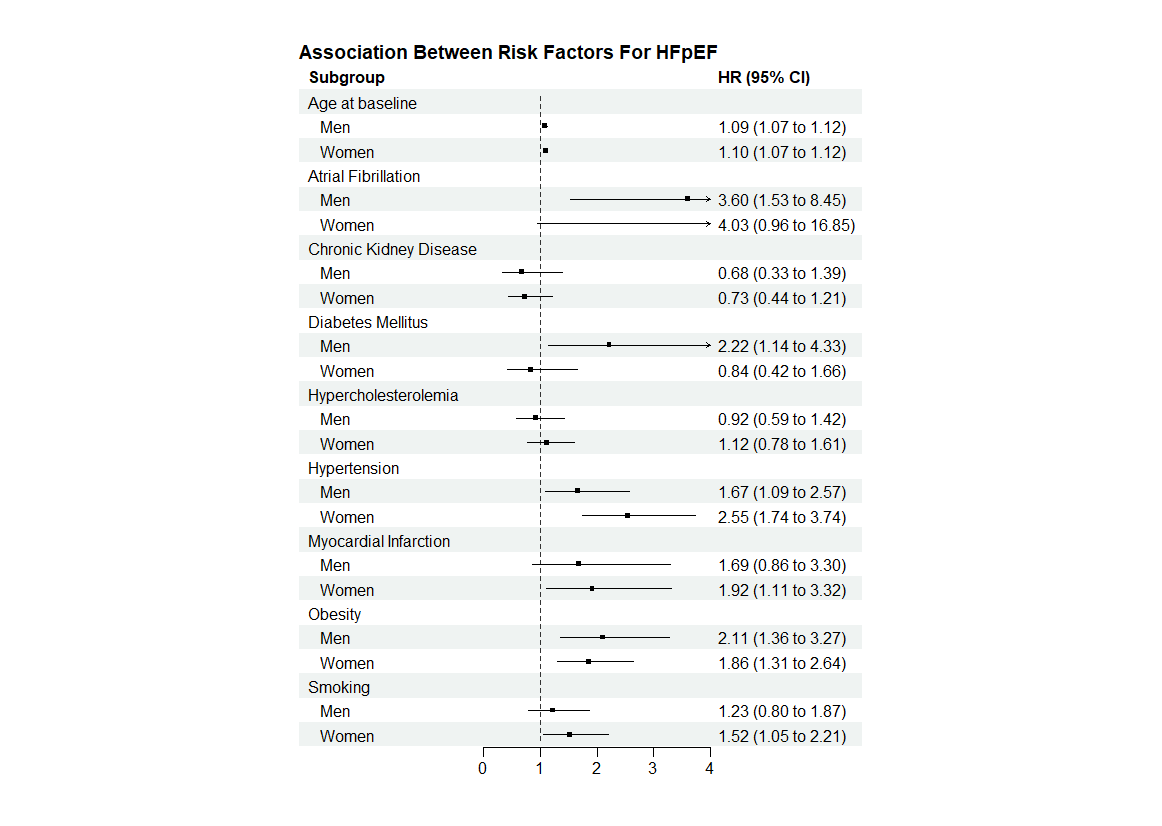
**

**Supplementary Figure 3: Population attributable fractions of eight comorbidities for heart failure with reduced (HFrEF) and heart failure with preserved ejection fraction (HFpEF).**

*PAF estimation was done using multivariable adjusted cause-specific Cox proportional hazard models adjusted for the other seven comorbidities and age at baseline.. PAFs are shown using HFrEF as dependent variable on the left (A) and HFpEF as dependent variable on the right (B).*

**
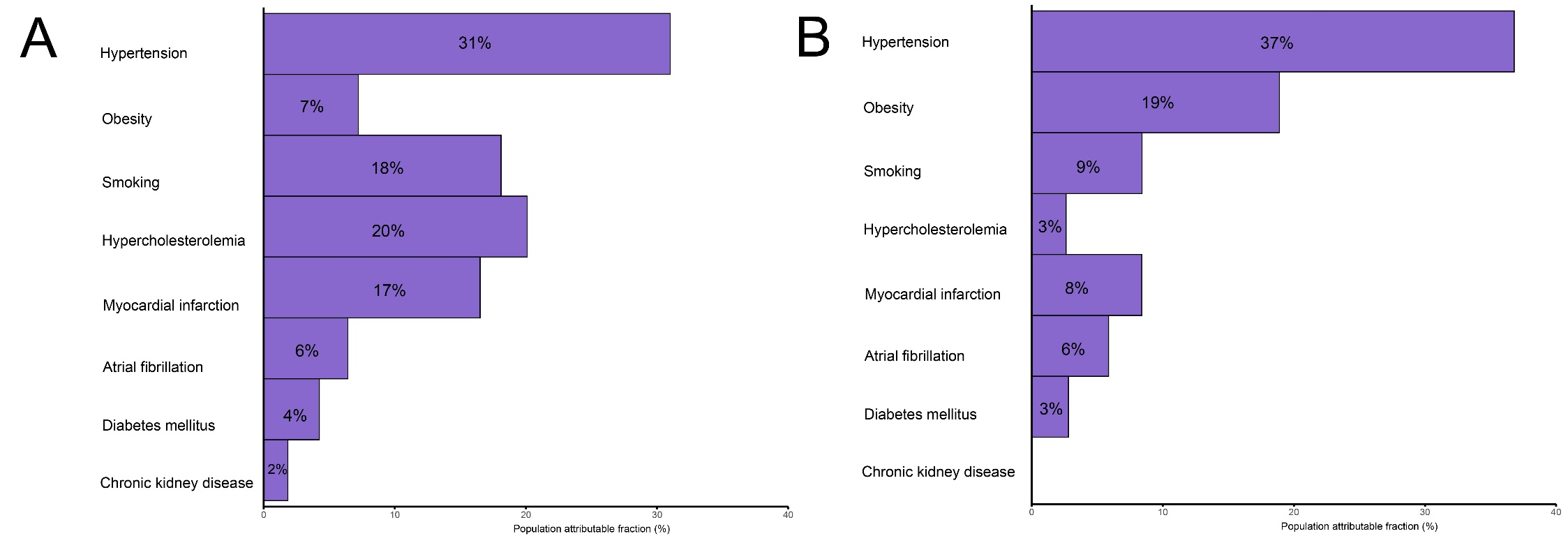
**
